# Local carbachol application induces oral microvascular recruitment and improves gastric tissue oxygenation during hemorrhagic shock in dogs

**DOI:** 10.3389/fimmu.2024.1369617

**Published:** 2024-03-19

**Authors:** Stefan Hof, Lara Lingens, Marius Michels, Carsten Marcus, Anne Kuebart, Anna Herminghaus, Inge Bauer, Olaf Picker, Richard Truse, Christian Vollmer

**Affiliations:** Department of Anesthesiology, Duesseldorf University Hospital, Duesseldorf, Germany

**Keywords:** tissue oxygenation, microcirculation, microvascular recruitment, hemorrhagic shock, carbachol, nitroglycerine, iloprost, local drug application

## Abstract

**Introduction:**

Hemorrhagic shock is characterized by derangements of the gastrointestinal microcirculation. Topical therapy with nitroglycerine or iloprost improves gastric tissue oxygenation but not regional perfusion, probably due to precapillary adrenergic innervation. Therefore, this study was designed to investigate the local effect of the parasympathomimetic carbachol alone and in combination with either nitroglycerine or iloprost on gastric and oral microcirculation during hemorrhagic shock.

**Methods:**

In a cross-over design five female foxhounds were repeatedly randomized into six experimental groups. Carbachol, or carbachol in combination with either nitroglycerine or iloprost were applied topically to the oral and gastric mucosa. Saline, nitroglycerine, or iloprost application alone served as control groups. Then, a fixed-volume hemorrhage was induced by arterial blood withdrawal followed by blood retransfusion after 1h of shock. Gastric and oral microcirculation was determined using reflectance spectrophotometry and laser Doppler flowmetry. Oral microcirculation was visualized with videomicroscopy. Statistics: 2-way-ANOVA for repeated measurements and Bonferroni post-hoc analysis (mean ± SEM; p < 0.05).

**Results:**

The induction of hemorrhage led to a decrease of gastric and oral tissue oxygenation, that was ameliorated by local carbachol and nitroglycerine application at the gastric mucosa. The sole use of local iloprost did not improve gastric tissue oxygenation but could be supplemented by local carbachol treatment. Adding carbachol to nitroglycerine did not further increase gastric tissue oxygenation. Gastric microvascular blood flow remained unchanged in all experimental groups. Oral microvascular blood flow, microvascular flow index and total vessel density decreased during shock. Local carbachol supply improved oral vessel density during shock and oral microvascular flow index in the late course of hemorrhage.

**Conclusion:**

The specific effect of shifting the autonomous balance by local carbachol treatment on microcirculatory variables varies between parts of the gastrointestinal tract. Contrary to our expectations, the improvement of gastric tissue oxygenation by local carbachol or nitroglycerine application was not related to increased microvascular perfusion. When carbachol is used in combination with local vasodilators, the additional effect on gastric tissue oxygenation depends on the specific drug combination. Therefore, modulation of tissue oxygen consumption, mitochondrial function or alterations in regional blood flow distribution should be investigated.

## 1 Introduction

Despite intensive research, acute traumatic hemorrhage remains a global burden, with a high mortality rate particularly within the first month after injury (1). During this period, the maintenance of intestinal tissue integrity seems to be crucial to reestablish global homeostasis and avoid secondary diseases (2). Unfortunately, hemorrhagic shock is characterized by microcirculatory failure, that is particularly pronounced within the gastrointestinal tract (3) leading to regional hypoxia and tissue injury. Therefore, there is need to detect therapeutic interventions that improve intestinal microcirculation during hemorrhagic shock. As there is a mismatch of macro- and microcirculatory variables in critically ill patients, the sole use of macrocirculatory measurements seems to be insufficient to guide the restoration of regional microcirculation during severe shock states (4). Meanwhile microcirculatory variables revealed to predict multi organ failure more sensitively than macrocirculatory variables in critically ill patients, highlighting the role of microvascular derangements in critically ill patients (5, 6).

There are two mechanisms mainly discussed leading to microvascular impairment during hemorrhagic shock within the gastrointestinal tract. First, excessive blood loss results in intravascular hypovolemia with reduced microvascular perfusion. Here the transfusion of blood products represents a therapeutic concept for hemorrhagic shock treatment (7, 8). Secondly, adrenergic vascular innervation and circulating catecholamines, aiming to ensure the maintenance of an adequate mean arterial perfusion pressure (9, 10), lead to regional vasoconstriction and thus to intestinal hypoperfusion and ischemia (11). The therapeutic approach to reopen collapsed vessels, increase total vessel density and enhance microvascular tissue oxygenation by pharmacological interventions is called microvascular recruitment (12, 13). From an integrated point of view, vascular tone is not only regulated by neurogenic innervation and circulating catecholamines, but is also influenced by regional metabolites like nitric oxide, adenosine and prostaglandins (14). Focusing on the metabolic part of microvascular blood flow regulation, Hilty et al. reported, that challenging the microcirculation by local nitroglycerine exposition is able to quantify the microvascular reserve in healthy volunteers (15), giving the perspective to deal with unphysiological conditions of restricted oxygen availability (16). This concept was successfully transferred into perioperative patients (17). Especially during acute heart failure the titration of low-dose nitroglycerine increases perfused capillary density (18), improves myocardial microcirculation and reduces cardiac filling pressure (19). Further, nitroglycerine reverts clinical manifestations of poor peripheral perfusion in patients undergoing circulatory shock (20). Likewise, iloprost, a prostacyclin analogue with vasodilative properties, demonstrated beneficial effects on microvascular variables and ameliorated endotoxemia-related acute kidney injury as a consequence of an improved tissue oxygenation in rats (21). Beside its potent effect on the microvasculature, iloprost also exerts beneficial effects on intestinal ischemia-reperfusion injury (22, 23) and reduces oxidative stress in a model of colitis in rodents (24).

In accordance with these findings, we successfully used local nitroglycerine and iloprost to increase gastric mucosal oxygenation during hemorrhagic shock in dogs in a previous study. In addition, nitroglycerine attenuated shock-induced impairment of the mucosal barrier integrity (25). Despite the well described vasodilative effect of both agents, regional perfusion markers remained unchanged after topical drug application (25). We supposed that precapillary adrenergic innervation during acute hemorrhage might counteract metabolic vasodilatation by nitric oxide and prostacyclin. Therefore, reducing adrenergic vasoconstriction could reveal as a possible target to maintain residual tissue perfusion. In previous studies, complete regional blockade of the sympathetic nervous system by epidural anesthesia revealed to maintain gastric tissue oxygenation under physiological conditions despite slightly compromised macrocirculatory variables. Unfortunately, peridural anesthesia exerted unfavorable effects on gastric tissue oxygenation during hemorrhagic shock (26, 27). This emphasizes the importance of at least partially preserved functionality of the sympathetic nervous system during hemorrhagic shock. Because the parasympathetic nervous system is the functional antagonist of adrenergic stimulation (28), the application of parasympathomimetic agents could restore the autonomic balance and maintain gastrointestinal perfusion without provoking disadvantageous effects of sympathetic blockade during acute hemorrhage. Therefore, this study was designed to investigate, if carbachol, a parasympathomimetic agent that acts as an agonist of muscarinic and nicotinic receptors, induces microvascular recruitment, improves oral and gastric microvascular perfusion and enhances tissue oxygenation in hemorrhagic dogs. Beside those microcirculatory considerations, carbachol is reported to improve intestinal barrier function by strengthening apical tight junctions and associated proteins during acute pancreatitis (29), lipopolysaccharide-induced intestinal damage (30) and ethanol-induced epithelial injury (31). Finally, carbachol induces intestinal mucus formation as a part of the physical barrier (32). In summary, these mechanisms including vascular and non-vascular effects could lead to reduced cell and tissue damage as reported during hypoxic challenges (33).

Whether the application of a parasympathomimetic is able to reestablish autonomous balance and leads to an increase of microvascular perfusion and tissue oxygenation during shock is unclear. Additionally, a parasympathomimetic agent in combination with nitroglycerine or iloprost might counteract precapillary adrenergic vasoconstriction and thus additionally improve microvascular flow. The systemic application of nitroglycerine, iloprost and carbachol could further diminish hemodynamic stability. In this context, our group reported the gastrointestinal microcirculation to be susceptible for topical therapies without systemic side effects during hemorrhagic shock (34–36). Thus, we studied the impact of local application of all substances.

The following study was designed to address the following questions:

(i) Does local application of the parasympathomimetic carbachol induce microvascular recruitment of oral microcirculation and increases gastric and oral tissue oxygenation during hemorrhagic shock?
(ii) Does additional local carbachol application further improve microcirculation, when applicated additionally to local nitroglycerine or iloprost treatment during acute hemorrhage?
(iii) Does local carbachol or the combination with either nitroglycerine or iloprost exert systemic side effects on circulatory variables?

## 2 Materials and methods

### 2.1 Animals and standard instrumentation

The data set was derived from five healthy female dogs (foxhounds, age: 3 – 10 years, weighing: 28 – 36 kg), that were treated repetitively in accordance with the NIH guidelines for animal care. All experiments were approved by the Local Animal Care and Use Committee (North Rhine- Westphalia State Agency for Nature, Environment, and Consumer Protection, Recklinghausen, Germany; ref. AZ 84-02.04.2012.A152). Animals were bred for experimental purposes and obtained from the animal research facility (ZETT, Zentrale Einrichtung für Tierforschung und wissenschaftliche Tierschutzaufgaben) of the Heinrich-Heine-University Duesseldorf. Food was withheld for 12 h prior to the experiments with free water access to ensure gastric depletion, since digestive activity might change gastrointestinal perfusion. Each dog underwent every experimental protocol in a randomized order and served as its own control (crossover design). Repeated experiments at the same individual reduce the total number of experiments needed to gain statistically significant results according to the 3R principle. Drug application was blinded to the investigators. After the experiments animals did not participate in any other experiment for at least three weeks to avoid carryover effects especially referring to the autonomous vegetative system. No animal was sacrificed during or after the experiments.

All experiments were performed under general anesthesia [induction of anesthesia: 4 mg·kg^-1^ propofol; maintenance of anesthesia: end-tidal sevoflurane 3.0 Vol % (37)] and mechanical ventilation [FiO_2_: 0.3, VT: 12.5 ml·kg^-1^ (38)]. No opioids were used due to the atraumatic instrumentation and lack of surgical stimulus and to avoid tissue protection via opioid receptors as side effect. In previous experiments we could show that systemic and regional hypercapnia improve microvascular oxygen saturation (3). Therefore, capnography was used to adjust respiratory rate to achieve normocapnia (etCO_2_: 35 mmHg). The left carotid artery was catheterized for invasive blood pressure measurement (Gould-Statham pressure transducers P23ID, Elk Grove, IL) and intermittent blood gas analysis (Rapidlab 860, Bayer AG, Germany). Both carotids were externalized before animals participated in the experiments to enable landmark guided arterial catheterization with low complication rates and a sufficient compression after the experiment for adequate hemostasis (39). All animals took part in previous experiments, so we did not perform surgical carotid externalization in direct relation to the described experiments. Transpulmonary thermodilution was used to determine cardiac output (CO [ml·kg^-1^·min^-1^]). In detail, the arterial catheter inserted to the carotid included a component to register the local temperature of arterial blood. (PiCCO 4.2 non US, PULSION Medical Systems, Munich, Germany). Every 30 minutes 10 ml of cold saline were injected intravenously via a peripheral vein at the forelimb. Alongside the bolus application, definitive temperature of the fluid bolus was registered by a thermistor that was connected to the intravenous catheter. After transpulmonary passage of the cold saline, the decrease of arterial blood temperature was measured in the arterial catheter and enables the calculation of CO. Systemic oxygen delivery (DO_2_ [ml·kg^-1^·min^-1^]) was calculated as total oxygen content of the blood multiplied by cardiac output. The heart rate (HR [1·min^-1^]) was measured by a spike-triggered electrocardiography (Powerlab, ADInstruments, Castle Hill, Austria). An orogastric tube was inserted to enable microvascular measurement at the gastric mucosa and local drug infusion. After the experiments animals were extubated and kept under direct supervision until they had completely recovered from anesthesia, including achieving an unassisted sufficient intake of wet food and water. Puncture sites were compressed to stop bleeding and examined to ensure adequate hemostasis. Then, the dogs were given back to their keepers in the animal research facility.

### 2.2 Experimental protocol

**Figure 1:**
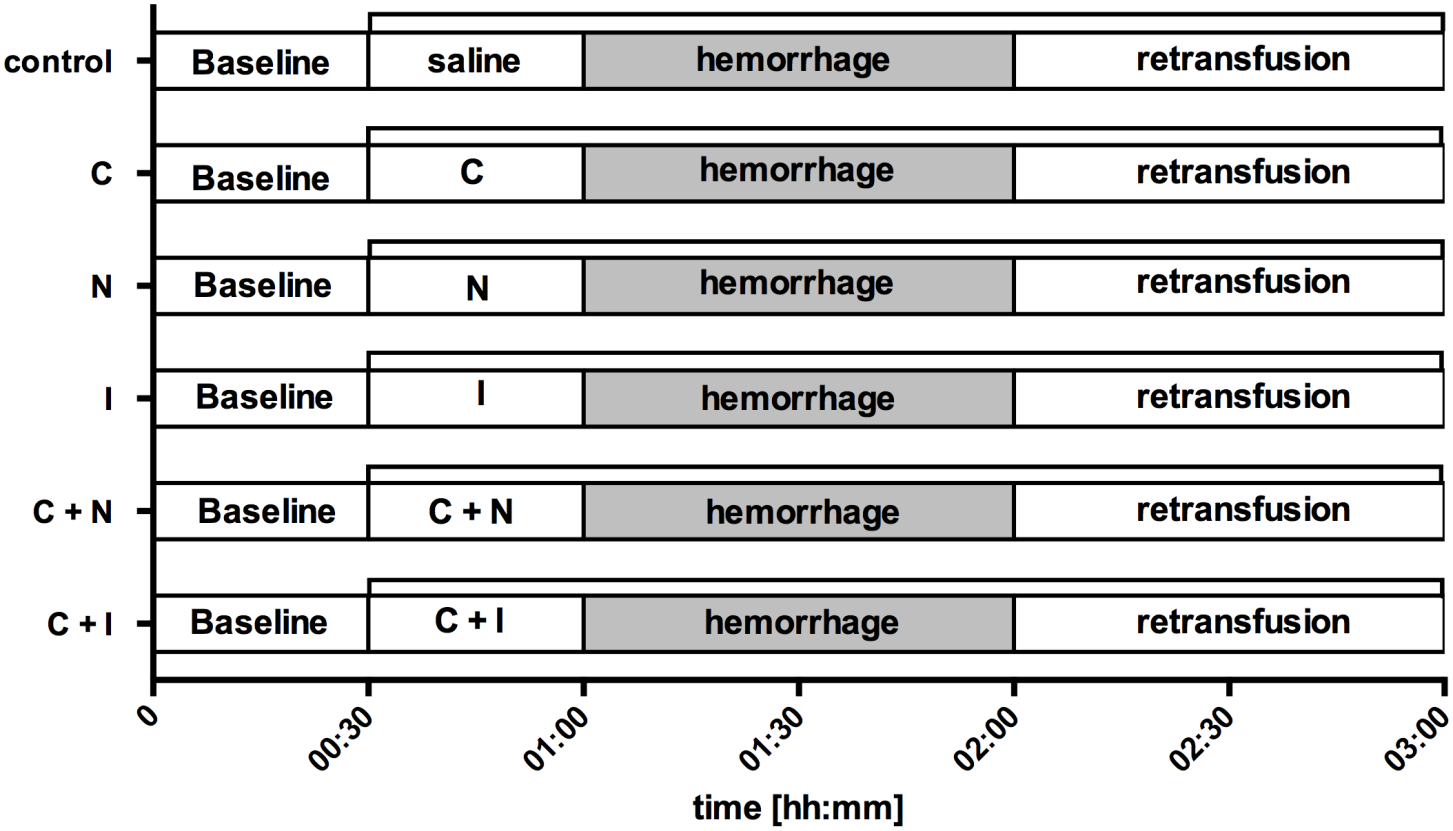
Experimental protocol. After the registration of baseline values (00:30) saline (control), carbachol (C), nitroglycerine (N), iloprost (I) or a combination of carbachol with either nitroglycerine (C + N) or iloprost (C + I) was applicated locally to the gastric and oral mucosa (01:00). The initial drug bolus was followed by a continuous rate for the rest of the experiments (∏). Then, hemorrhagic shock was induced by arterial blood withdrawal and measurements were performed after 30 min and 60 min to assess early (01:30) and late (02:00) shock effects. The period of acute hemorrhage is marked grey in this figure. Finally, the removed blood volume was retransfused and measurements were obtained during early (02:30) and late (03:00) course of retransfusion. Time is indicated as [hh:mm].

After the induction of general anesthesia, intubation and standardized instrumentation, baseline values were recorded under steady state conditions and animals were randomized to one of six experimental protocols (**Figure 1**). Steady state conditions were defined as stability of hemodynamic variables (HR [1·min^-1^] and MAP [mmHg]), microcirculatory measurements (reflectance spectrophotometry) and ventilation variables (etCO_2_ [mmHg], etSevo [Vol%]). First, an initial drug bolus was applicated simultaneously to the oral region and the gastric cave as described below. Then, continuous drug infusion was started. After thirty minutes of observation, a fixed-volume hemorrhage was inducted by removing 20% of the estimated total blood volume within 5 min duration via an intravenous peripheral cannula and the arterial catheter (40). According to Advanced Trauma Life Support (ATLS) shock classification, this represents a class II shock (41). Shock was held for one hour without further correction, then the heparinized shed blood was retransfused using a conventional transfusion set with a 200 µm filter. Afterwards, animals were observed for one hour, then experiments were stopped, and animals were led back to spontaneous breathing and consciousness.

#### 2.2.1 Interventional groups

Experimental groups differed according to the combination of nitroglycerine (Nitrolingual infusion, G. Pohl-Boskamp & co KG, Hohenlockstedt, Germany), iloprost (5-cis Iloprost, Cayman Chemicals, Ann Arbor, MI, USA), carbachol (Carbamylcholine chloride, Sigma-Aldrich Corporation, St. Louis, USA) and saline application. All investigators were blinded to the pharmacological treatment. The experimental protocol of acute hemorrhage and retransfusion did not differ between the groups. All experimental groups - including those with a sole nitroglycerine or iloprost treatment - have been performed exclusively for the here addressed research questions and have not been published before.

#### 2.2.2 Saline (control)

Control animals did not receive drug application prior to acute hemorrhage. Instead, a local application of saline was used to exclude volume effects. After an initial gastric bolus of 0.25 ml·kg^-1^ saline, a continuous fluid supplementation was generated by the application of 2 ml·kg^-1^·h^-1^ at the gastric mucosa and 0.66 ml·kg^-1^·h^-1^ at the oral mucosa. Total fluid substitution by gastric and oral drug application was equally in all experimental groups.

#### 2.2.3 Nitroglycerine (N)

Animals received an initial gastric nitroglycerine bolus of 25 µg·kg^-1^ followed by 500 µg·kg^-1^·h^-1^ continuous nitroglycerine application at the gastric mucosa and 165 µg·kg^-1^·h^-1^ continuous nitroglycerine application at the oral mucosa. The nitroglycerine dose used in this study was reported to improve gastric µHbO_2_ in a previous study (25).

#### 2.2.4 Iloprost (I)

Animals received an initial gastric iloprost bolus of 100 ng·kg^-1^ body weight followed by 2 µg·kg^-1^·h^-1^ continuous iloprost application at the gastric mucosa and 0.66 µg·kg^-1^·h^-1^ continuous iloprost application at the oral mucosa. The iloprost dose used in this study was reported to improve gastric µHbO_2_ in a previous study (25).

#### 2.2.5 Carbachol (C)

Animals received an initial gastric carbachol bolus of 20 µg·kg^-1^ body weight followed by 10 µg·kg^-1^·h^-1^ continuous carbachol application at the gastric mucosa and 3.33 µg·kg^-1^·h^-1^ continuous carbachol application at the oral mucosa. The carbachol dose used in this study is adapted to recommendations for the therapeutic approach of cats and dogs (42).

#### 2.2.6 Nitroglycerine + Carbachol (N + C)

To investigate the additional effect of topical carbachol application on nitroglycerine treatment, animals received a gastric bolus of 25 µg·kg^-1^ nitroglycerine and 20 µg·kg^-1^ carbachol. Gastric (nitroglycerine: 500 µg·kg^-1^·h^-1^; carbachol: 10 µg·kg^-1^·h^-1^) and oral (nitroglycerine 165 µg·kg^-1^·h^-1^; carbachol: 3.33 µg·kg^-1^·h^-1^) infusion were performed according to the protocol in groups with single drug treatment.

#### 2.2.7 Iloprost + Carbachol (I + C)

To investigate the additional effect of topical carbachol application on iloprost treatment, animals received a gastric bolus of 100 ng·kg^-1^ iloprost and 20 µg·kg^-1^ carbachol. Gastric (iloprost: 2 µg·kg^-1^·h^-1^; carbachol: 10 µg·kg^-1^·h^-1^) and oral (iloprost 0.66 µg·kg^-1^·h^-1^; carbachol: 3.33 µg·kg^-1^·h^-1^) infusion were performed according to the protocol in groups with single drug treatment.

### 2.3 Measurements

#### 2.3.1 Reflectance spectrophotometry and laser Doppler flowmetry

Gastric and oral microcirculation were evaluated using a combined measurement of two analytic principles: reflectance spectrophotometry and laser Doppler flowmetry (O2C, LEA Medizintechnik, Gießen, Germany). As described previously, white light with a spectrum between 450 nm und 1000 nm wavelength and a diode generated pulse laser (CW-mode, 820 nm) was emitted into the subepithelial tissue of gastric and oral mucosa. Dependent on its oxygen saturation grade, hemoglobin absorbs different wavelengths of white light. Therefore, the averaged hemoglobin saturation can be calculated from the scattered and reflected, complementary light spectrum. Since blood is not distributed equally within the microcirculatory compartment with a predominantly venous proportion and tissue reflectance spectrophotometry is not able to distinguish between arterial and venous vessels, measurements report mainly postcapillary oxygen saturation (µHbO_2_). According to the principle of last meadow, µHbO_2_ is a marker to determine tissue oxygen reserve under conditions of generally maintained microvascular vessel architecture. Not focusing on the spectral components, but on the total amount of reflected light, relative hemoglobin content (rHb) can be calculated. Thereby, relative hemoglobin content refers to a predetermined volume of the targeted tissue and correlates antiproportionally to the intensity of reflected light. Vessels that are not part of the microcirculatory compartment lead to total light absorption in consequence of the extensive hemoglobin load in larger vessels.

Laser-Doppler flowmetry is based on the frequency shift, that is generated at the surface of moving particles. Since microcirculatory vessels form a tridimensional network, complex algorithms are used to transform the reflected laser signal and results are reported in arbitrary units [aU] (43). All values reported are the average of measurements within 5 min.

#### 2.3.2 Oral incident dark-field imaging

IDF-imaging is based on initial considerations of Sherman et al. and uses epi-illumination to visualize the microcirculation (44). Further improvement of the technical devices and slight changes in the principle of measurement led to a high-resolution handheld camera called Cytocam (Braedius Medical, Huizen, The Netherlands), which is declared to be the third generation videomicroscope for noninvasive microcirculatory assessment. This device is recommended by an international expert group of the European Society of Intensive Care Medicine (ESICM) to evaluate the microcirculation (11). Conceptionally, pulsed green light is emitted to the targeted tissue and reflected by interstitial cells and matrix components. A complex lens system at the tip of the Cytocam device then creates short video sequences of the reflected light. Since light with a wavelength of 530 nm meets the isosbestic point of oxygenated and deoxygenated hemoglobin, illumination of erythrocytes as the main hemoglobin carriers leads to total absorption. Erythrocytes during the tissue passage then occur as dark light recesses and form the contour of microvascular vessels. The videos were recorded according to the quality requirements of an international expert round and saved anonymized for blinded analysis. Total vessel density (TVD) as a marker of microvascular recruitment and perfused vessel density (PVD) as well as the proportion of perfused vessels (PPV) as indices of microvascular perfusion were determined using an automatic analyzing software. Further, the microvascular flow index was calculated to access the predominant microvascular blood flow characteristics. In detail, the screen is divided into four squares and flow is characterized as not existent (scoring = 0), intermittent (scoring = 1), sluggish (scoring = 2) or regular (scoring = 3). To avoid sampling errors, five videos were recorded, and averaged values are reported.

### 2.4 Statistics

Macro- and microcirculatory data were obtained during the last 5 min of each intervention period under steady-state conditions and are presented as absolute values of mean ± standard error (mean ± SEM) for five dogs. Normal data distribution was assessed in Q-Q-plots (IBM SPSS Statistics, International Business Machine Corp., United States). A two-way analysis of variance for repeated measurements (ANOVA) and a Bonferroni test as *post-hoc* test for statistical analysis (GraphPad Prism version 6.05 for Windows, GraphPad Software, La Jolla California United States) were used to determine differences within and between the experimental groups. p < 0.05 is considered as statistically significant. Multiple measurements enable metric statistical testing of microvascular flow index and heterogeneity index as semiquantitative scores that generally require non-parametric analysis (3).

## 3 Results

### 3.1 Effect of local drug application on gastric microcirculation

**Figure 2:**
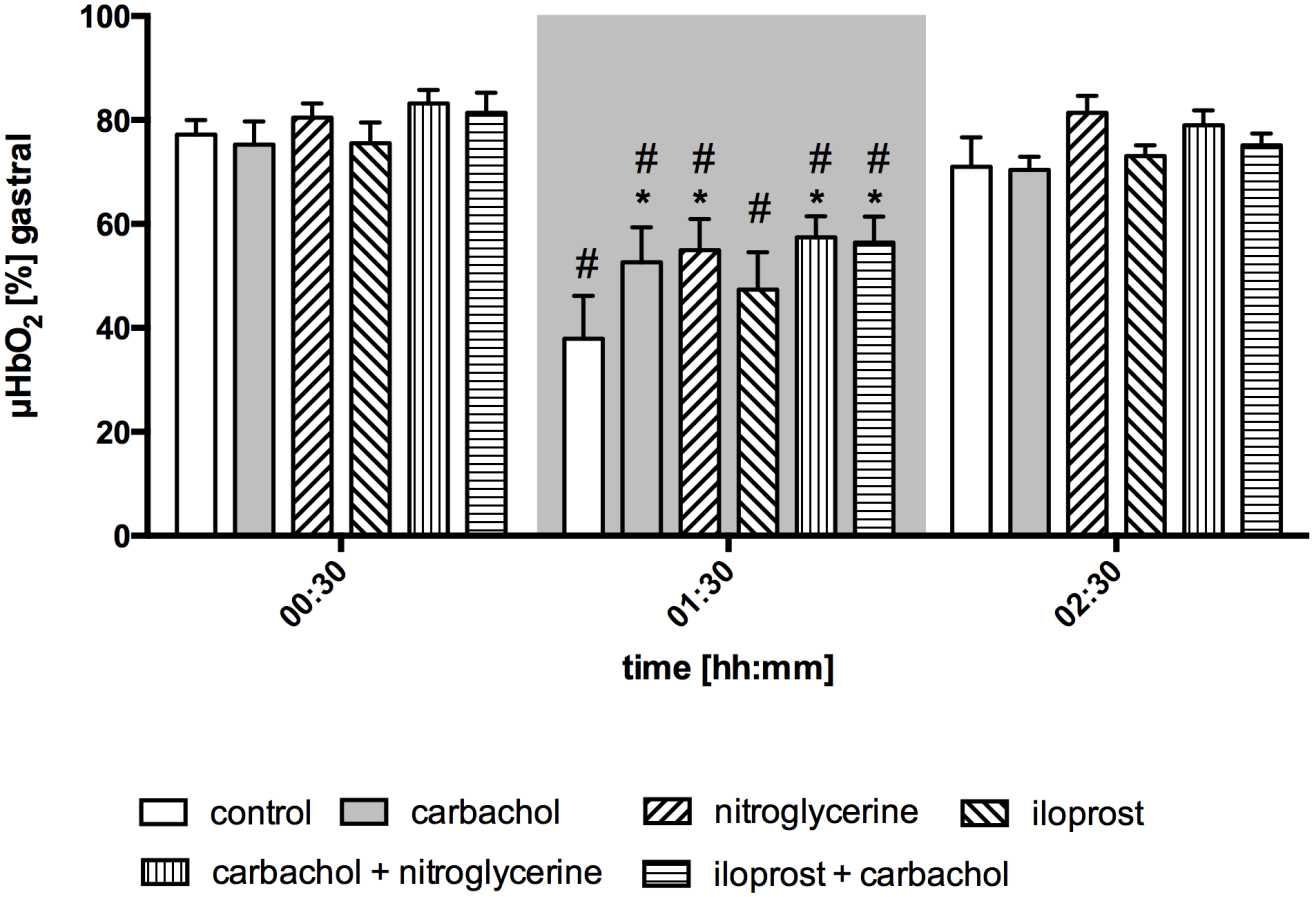
Postcapillary oxygen saturation of gastric mucosa. Postcapillary oxygen saturation (µHBO_2_) of the gastric mucosa in percentage points (%) under baseline conditions (00:30), during early hemorrhagic shock (01:30) and after retransfusion (02:30) of the shed blood. The period of hemorrhagic shock is visualized by a grey box. Local application with either a sole carbachol, nitroglycerine or iloprost or a combined treatment with nitroglycerine or iloprost and carbachol are marked according to the symbols shown in the legend. The time is indicated as [hh:mm]. Data are presented as mean ± SEM for n = 5 female dogs; * p < 0.05 between the indicated experimental group and the control group at one time point. # p < 0.05 vs. baseline value. 2-way ANOVA for repeated measurements followed by the Bonferroni *post-hoc* test.

Gastric µHbO_2_ did not differ between experimental groups under baseline conditions and remained unchanged after local drug application. The induction of hemorrhagic shock led to a pronounced decrease of µHbO_2_ that was diminished by local nitroglycerine and carbachol application (**Figure 2**) but not with iloprost. Combining carbachol and nitroglycerine did not further increase µHbO_2_, whereas adding carbachol to iloprost significantly raised µHbO_2_ during acute hemorrhagic shock. Whereas µHbO_2_ after 1 h of hemorrhagic shock increased in animals that received nitroglycerine, the beneficial effect of local carbachol application was limited to the initial shock (**Table 1**). Across all groups, µHbO_2_ returned to baseline values after retransfusion of the shed blood. Gastric µflow and rHb remained stable over the time in all experimental groups. There were no differences in gastric µflow and rHb values referring to the experimental groups (**Table 1**).

**Table 1:** Gastric microcirculation.

Variable	group	00:30				01:00				01:30				02:00				02:30				03:00			
µHbO_2_ gastral [%]	control	77	±	3		74	±	6		38	±	8	#	45	±	9	#	71	±	6		75	±	5	
	C	75	±	4		76	±	2		53	±	7	#*	58	±	5	#	70	±	3		78	±	2	
	N	81	±	3		83	±	2		55	±	6	#*	61	±	8	#*	81	±	3		80	±	2	
	I	76	±	4		81	±	3		47	±	7	#	53	±	7	#	73	±	2		75	±	4	
	C + N	83	±	3		84	±	2		57	±	4	#*	56	±	7	#	79	±	3		81	±	4	
	C + I	81	±	4		83	±	4		56	±	5	#*	58	±	3	#	75	±	2		80	±	1	
µflow gastral [aU]	control	154	±	29		170	±	9		128	±	10		175	±	21		191	±	18		214	±	26	
	C	177	±	11		195	±	42		121	±	19		117	±	17		156	±	31		141	±	32	
	N	187	±	26		201	±	26		145	±	21		190	±	32		213	±	31		186	±	23	
	I	127	±	24		194	±	21		169	±	31		171	±	31		167	±	34		153	±	18	
	C + N	110	±	14	§	170	±	24		101	±	13		115	±	11		165	±	22		134	±	31	
	C + I	129	±	11		170	±	21		133	±	14		147	±	24		186	±	23		175	±	30	
µvelo gastral [aU]	control	19	±	1		18	±	1		16	±	1		19	±	1		20	±	1		22	±	2	
	C	19	±	1		22	±	3		16	±	2		16	±	1		19	±	2		20	±	2	
	N	21	±	2		21	±	2		17	±	1		20	±	2		22	±	3		21	±	3	
	I	16	±	2		19	±	2		16	±	1		18	±	3		17	±	2		18	±	2	
	C + N	15	±	1	§	19	±	2		14	±	1		14	±	1	§	18	±	1		17	±	2	
	C + I	18	±	1		20	±	2		18	±	1		18	±	2		21	±	2		21	±	3	
rHb gastral [aU]	control	56	±	6		54	±	6		47	±	8		45	±	6		54	±	5		55	±	6	
	C	49	±	5		54	±	7		52	±	5		50	±	4		43	±	5		44	±	7	
	N	60	±	5		64	±	4		55	±	4		54	±	3		64	±	6		59	±	6	
	I	49	±	6		54	±	7		42	±	2		44	±	5		43	±	5		47	±	7	
	C + N	55	±	7		61	±	6		47	±	7		52	±	6		53	±	9		51	±	8	
	C + I	52	±	6		61	±	5		50	±	5		51	±	6		54	±	6		56	±	6	

Postcapillary oxygen saturation (µHbO2; [%]), Microvascular blood flow (µflow; [aU]), microvascular blood flow velocity (µvelo; [aU]) and relative hemoglobin content (rHb; [aU]) of gastric mucosa after a sole or combined local treatment with carbachol (C), nitroglycerine (N) or iloprost (I). The time is indicated as [hh:mm]. Values are reported under baseline conditions (00:30), after drug application (01:00), during early (01:30) and late (02:00) hemorrhage and during early (02:30) and late blood retransfusion (03:00). Data are presented as mean ± SEM for n = 5 female dogs. # p < 0.05 vs. baseline values; * p < 0.05 vs. control group; § p < 0.05 vs. a sole nitroglycerine or iloprost treatment, if carbachol was applicated simultaneously. 2-way ANOVA for repeated measurements followed by Bonferroni post-hoc test.

### 3.2 Effect of local drug application on oral microcirculation

**Figure 3:**
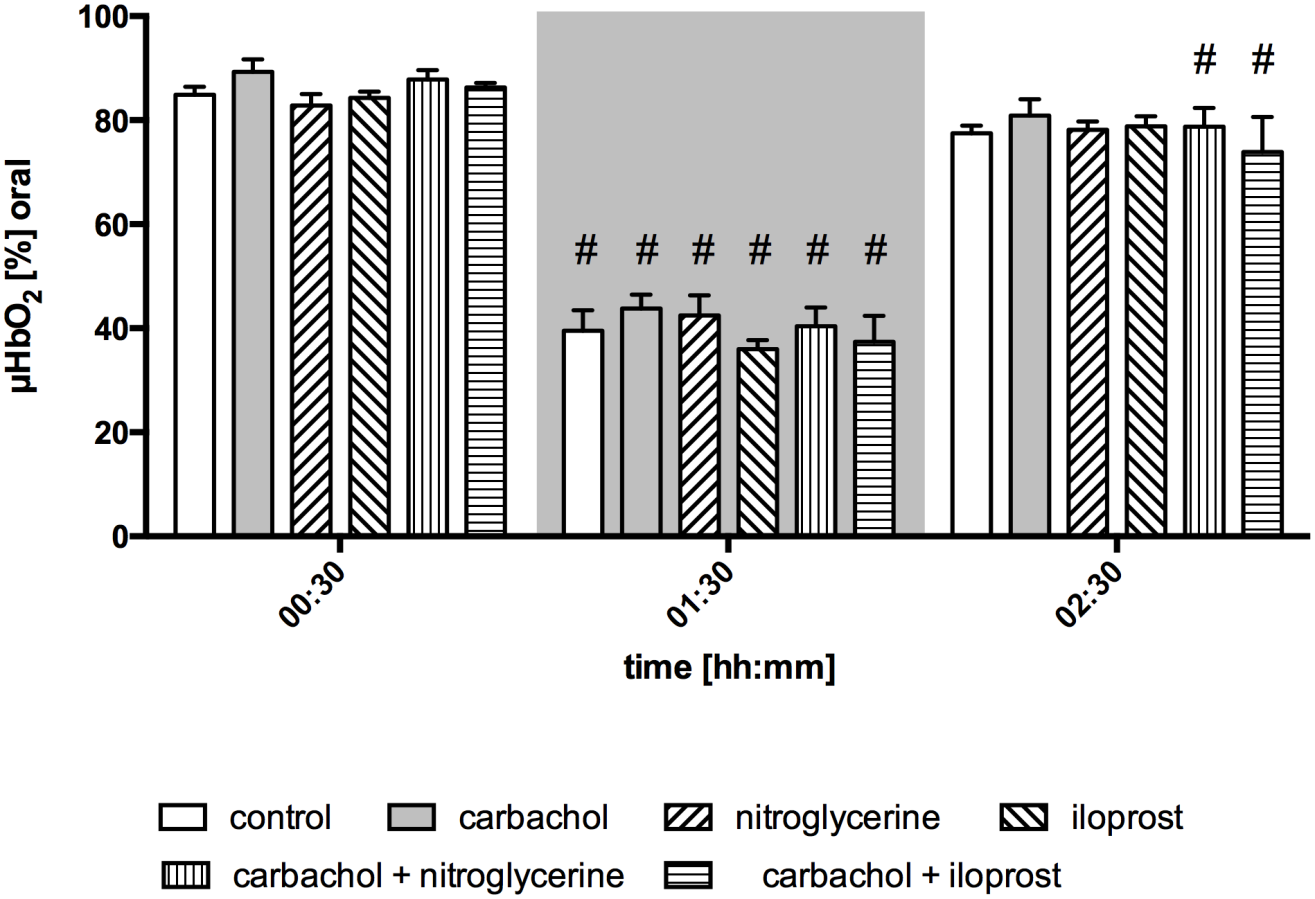
Postcapillary oxygen saturation of oral mucosa. Postcapillary oxygen saturation (µHBO_2_) of oral mucosa in percentage points (%) under baseline conditions (00:30), during early hemorrhagic shock (01:30) and after retransfusion (02:30) of the shed blood. The period of hemorrhagic shock is visualized by a grey box. Local application with either a sole carbachol, nitroglycerine or iloprost or a combined treatment with nitroglycerine or iloprost and carbachol are marked according to the symbols shown in the legend. The time is indicated as [hh:mm]. Data are presented as mean ± SEM for n = 5 female dogs; # p < 0.05 vs. the individual baseline value. There were no differences between the experimental groups at one time point. 2-way ANOVA for repeated measurements followed by the Bonferroni *post-hoc* test.

**Figure 4:**
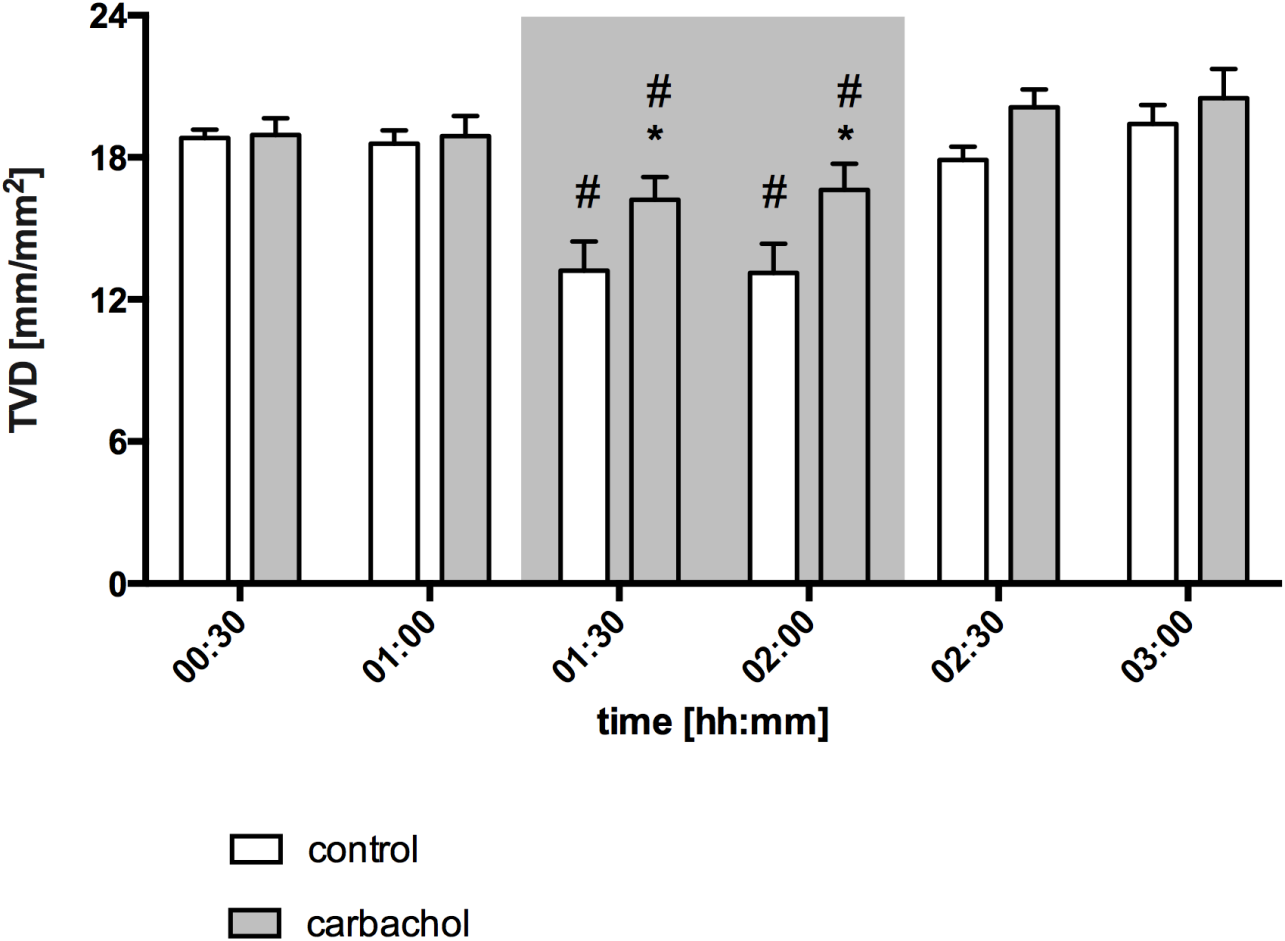
Total vessel density of oral mucosa after local carbachol treatment. Total vessel density (TVD) of oral mucosa in mm/mm^2^. The period of hemorrhagic shock is visualized by a grey box. Local application with either saline or carbachol is marked according to the symbols shown in the legend. Other experimental groups are not shown for reasons of clarity. The time is indicated as [hh:mm]. Data are presented as mean ± SEM for n = 5 female dogs; * p < 0.05 between animals, that received saline or carbachol at one time point. 2-way ANOVA for repeated measurements followed by the Bonferroni *post-hoc* test.

**Figure 5:**
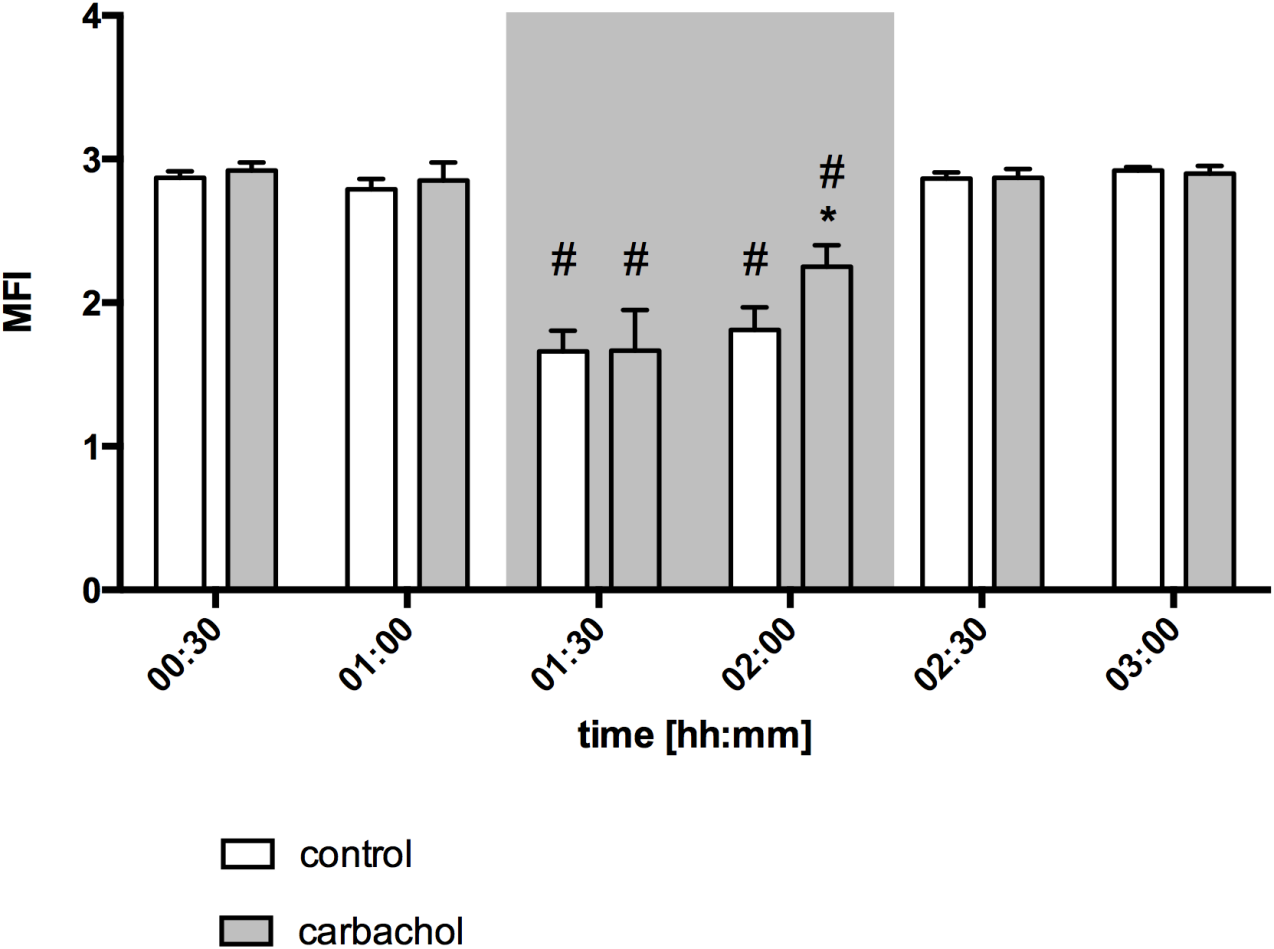
Microvascular flow index of oral mucosa after local carbachol treatment: Microvascular flow index (MFI) of oral mucosa. The period of hemorrhagic shock is visualized by a grey box. Local application with either saline or carbachol is marked according to the symbols shown in the legend. Other experimental groups are not shown for reasons of clarity. The time is indicated as [hh:mm]. Data are presented as mean ± SEM for n = 5 female dogs; * p < 0.05 between animals, that received saline or carbachol at one time point. 2-way ANOVA for repeated measurements followed by the Bonferroni *post-hoc* test.

Baseline values of oral µHbO_2_, µflow and rHb did not differ between experimental groups. Similar to gastric measurements, the induction of hemorrhagic shock led to a significantly decrease of µHbO_2_ at the oral mucosa. Neither the local application of carbachol nor the local application of nitroglycerine or iloprost influenced oral µHbO_2_ during acute hemorrhage. Furthermore, the combination of local carbachol and nitroglycerine application as well as the combination of local carbachol and iloprost application failed to improve mucosal µHbO_2_ during acute hemorrhage (**Figure 3**). Retransfusion of the taken blood reestablished initial µHbO_2_-values at the end of experiments (**Table 2**). Oral µflow and rHb decreased after shock induction in control animals. None of the treatments improved µflow or rHb during hemorrhagic shock (**Table 2**). TVD at baseline measurement was similar in all experimental groups. The induction of hemorrhagic shock led to a significant decrease of TVD in all experimental groups. Only the local application of carbachol improved TVD during acute hemorrhage (**Figure 4**). Initial values of PVD and MFI did not vary between experimental groups. PVD decreased during hemorrhagic shock and was not affected by local drug application. Similarly, MFI decreased in control animals after the induction of acute hemorrhagic shock. After 1 h of the ongoing hemorrhage, MFI was higher in animals that received carbachol compared to controls (**Figure 5**) and to animals with nitroglycerine or iloprost treatment. PVD and MFI values reached baseline level after retransfusion of the taken blood in all experimental groups (**Table 2**).

**Table 2:** Oral microcirculation.

Variable	group	00:30				01:00				01:30				02:00				02:30				03:00			
µHbO_2_ oral [%]	control	85	±	2		77	±	2		40	±	4	#	44	±	4	#	77	±	1		88	±	2	
	C	89	±	2		86	±	2		44	±	3	#	50	±	3	#	81	±	3		90	±	3	
	N	83	±	2		82	±	4		43	±	4	#	51	±	3	#	78	±	2		88	±	2	
	I	84	±	1		83	±	1		36	±	2	#	48	±	3	#	79	±	2		90	±	4	
	C + N	88	±	2		78	±	2		40	±	4	#	45	±	2	#	79	±	4	#	87	±	2	
	C + I	86	±	1		82	±	4		37	±	5	#	41	±	5	#	74	±	7	#	88	±	3	
µflow oral [aU]	control	121	±	18		72	±	5		39	±	14	#	31	±	7	#	83	±	7		154	±	25	
	C	174	±	19		145	±	26	*	34	±	10	#	40	±	4	#	160	±	35	*	179	±	49	
	N	161	±	30		179	±	36	*	42	±	7	#	36	±	11	#	122	±	36		150	±	25	
	I	172	±	19		148	±	20	*	34	±	8	#	34	±	7	#	121	±	21		193	±	25	
	C + N	158	±	21		176	±	43	*	37	±	6	#	42	±	9	#	119	±	19		176	±	47	
	C + I	148	±	28		129	±	27		57	±	13	#	69	±	20	#	147	±	24		196	±	21	
µvelo oral [aU]	control	24	±	3		19	±	1		15	±	3	#	13	±	1	#	20	±	1		29	±	3	
	C	27	±	2		25	±	3		14	±	2	#	16	±	2	#	29	±	3	*	29	±	5	
	N	27	±	3		31	±	3	*	18	±	3	#	13	±	2	#	24	±	4		26	±	2	
	I	28	±	2		27	±	2		13	±	2	#	13	±	1	#	23	±	2		31	±	4	
	C + N	25	±	2		29	±	5	*	14	±	1	#	16	±	2	#	24	±	3		29	±	5	
	C + I	26	±	4		25	±	5		20	±	4		25	±	5	*§	30	±	4	*	31	±	2	
rHb oral [aU]	control	96	±	3		90	±	2		60	±	5	#	62	±	3	#	84	±	4	#	89	±	2	
	C	101	±	2		98	±	2		68	±	2	#	73	±	6	#*	85	±	4	#	93	±	4	
	N	98	±	3		96	±	2		70	±	3	#	76	±	4	#*	86	±	3	#	93	±	2	
	I	98	±	2		93	±	4		58	±	4	#	68	±	5	#	89	±	4	#	90	±	7	
	C + N	99	±	3		100	±	2		69	±	4	#	70	±	6	#	87	±	6	#	93	±	4	
	C + I	97	±	3		94	±	2		60	±	5	#	64	±	4	#	82	±	5	#	94	±	3	
TVD oral [mm·mm^-2^]	control	19	±	0		19	±	1		13	±	1	#	13	±	1	#	18	±	1		19	±	1	
	C	19	±	1		19	±	1		16	±	1	#*	17	±	1	#*	20	±	1		21	±	1	
	N	19	±	1		19	±	1		14	±	2	#	14	±	1	#	19	±	0		19	±	1	
	I	20	±	1		19	±	1		13	±	2	#	14	±	2	#	18	±	1		20	±	1	
	C + N	19	±	1		19	±	1		15	±	1	#	14	±	1	#	19	±	1		20	±	1	
	C + I	18	±	1		18	±	2		13	±	1	#	14	±	2	#	19	±	2		19	±	1	
PVD oral [mm·mm^-2^]	control	12	±	1		11	±	1		5	±	1	#	5	±	1	#	9	±	2	#	11	±	1	
	C	12	±	1		13	±	1		8	±	1	#	9	±	1	*	14	±	1	*	14	±	1	
	N	12	±	1		12	±	1		6	±	1	#	6	±	1	#	11	±	1		12	±	1	
	I	13	±	2		11	±	1		6	±	1	#	7	±	1	#	11	±	1		12	±	1	
	C + N	11	±	1		12	±	1		6	±	1	#	5	±	1	#	10	±	1		12	±	2	
	C + I	10	±	3		11	±	3		6	±	2	#	7	±	3		10	±	3		12	±	2	
PPV oral [%]	control	62	±	2		59	±	3		40	±	7	#	38	±	7	#	47	±	10		58	±	5	
	C	60	±	5		67	±	4		46	±	6		48	±	4		66	±	4	*	67	±	3	
	N	64	±	2		65	±	3		42	±	6	#	45	±	4	#	59	±	5		59	±	5	
	I	64	±	6		55	±	5		43	±	5	#	45	±	4	#	58	±	4		61	±	4	
	C + N	54	±	4		57	±	2		40	±	5		36	±	5	#	52	±	7		55	±	11	
	C + I	54	±	13		56	±	13		41	±	10		47	±	11		52	±	10		57	±	9	
MFI oral	control	2,9	±	0,1		2,8	±	0,1		1,7	±	0,1	#	1,8	±	0,2	#	2,9	±	0,0		2,9	±	0,0	
	C	2,9	±	0,1		2,9	±	0,1		1,7	±	0,3	#	2,3	±	0,2	#*	2,9	±	0,1		2,9	±	0,1	
	N	2,8	±	0,1		2,9	±	0,1		1,5	±	0,3	#	1,7	±	0,2	#	2,8	±	0,1		2,8	±	0,1	
	I	2,8	±	0,1		2,8	±	0,1		1,6	±	0,2	#	1,6	±	0,2	#	2,7	±	0,1		2,9	±	0,1	
	C + N	2,9	±	0,1		3,0	±	0,0		1,6	±	0,3	#	1,8	±	0,2	#	2,8	±	0,1		2,9	±	0,1	
	C + I	2,8	±	0,1		2,8	±	0,2		1,5	±	0,2	#	2,0	±	0,2	#§	2,8	±	0,1		2,9	±	0,1	

Postcapillary oxygen saturation (µHbO2; [%]), Microvascular blood flow (µflow; [aU]), microvascular blood flow velocity (µvelo; [aU]), relative hemoglobin content (rHb; [aU]), total vessel density (TVD; [mm/mm2]), perfused vessel density (PVD; [mm/mm2]), proportion of perfused vessels (PPV; [mm/mm2]) and microvascular flow index (MFI) of gastric mucosa after a sole or combined local treatment with carbachol (C), nitroglycerine (N) or iloprost (I). Values are reported under baseline conditions (00:30), after drug application (01:00), during early (01:30) and late (02:00) hemorrhage and during early (02:30) and late blood retransfusion (03:00). The time is indicated as [hh:mm]. Data are presented as mean ± SEM for n = 5 female dogs. # p < 0.05 vs. baseline values; * p < 0.05 vs. control group; § p < 0.05 vs. a sole nitroglycerine or iloprost treatment, if carbachol was applicated simultaneously. 2-way ANOVA for repeated measurements followed by Bonferroni post-hoc test.

### 3.3 Effect of local drug application on systemic circulatory variables

Baseline values of CO and DO_2_ did not differ between experimental groups. The induction of acute hemorrhagic shock led to a pronounced decrease of CO and DO_2_. Retransfusion reestablished adequate CO and DO_2_. Local drug application had no effect on systemic circulatory variables (**Table 3**). Compared to baseline values plasmatic lactate levels increased in all groups with local carbachol application during hemorrhagic shock and remained elevated after blood retransfusion for the rest of the experiments. The cotreatment with nitroglycerine or iloprost mitigated this effect of local carbachol supply on lactate levels during the retransfusion period. At the end of the experiments only the carbachol group had increased lactate levels compared to the control group. Animals that received a sole nitroglycerine or iloprost treatment revealed a slight increase of plasmatic lactate levels, that did not exceed the physiological range. Slight changes of arterial blood gases, pH-value and bicarbonate concentration remained within the physiological range in all experimental groups (**Table 4**).

**Table 3:** Macrocirculatory variables.

Variable	group	00:30				01:00				01:30				02:00				02:30				03:00			
DO_2_ [ml·kg^-1^·min^-1^]	control	14	±	1		13	±	1		8	±	0	#	8	±	0	#	14	±	1		14	±	1	
	C	15	±	1		14	±	1		8	±	1	#	9	±	1	#	14	±	1		15	±	1	
	N	14	±	1		13	±	1		7	±	1	#	9	±	0	#	14	±	1		13	±	1	
	I	14	±	1		14	±	0		7	±	0	#	9	±	1	#	14	±	1		14	±	1	
	C + N	14	±	0		13	±	1		8	±	0	#	8	±	0	#	14	±	1		14	±	1	
	C + I	15	±	1		14	±	1		8	±	1	#	9	±	1	#	15	±	1		15	±	1	
SVR [mmHg·l^-1^·min]	control	22	±	1		24	±	1		31	±	2	#	31	±	2	#	29	±	2	#	24	±	2	
	C	21	±	1		22	±	1		33	±	4	#	32	±	3	#	28	±	1	#	23	±	1	
	N	23	±	2		22	±	2		30	±	2	#	28	±	1	#	24	±	1		22	±	1	
	I	21	±	1		22	±	1		33	±	3	#	31	±	2	#	29	±	2	#	24	±	3	
	C + N	21	±	2		21	±	2		25	±	3	#*§	29	±	3	#*	22	±	1		20	±	1	
	C + I	22	±	1		23	±	2		30	±	2	#	31	±	1	#	28	±	1	#	22	±	1	
CO [ml·kg^-1^·min^-1^]	control	81	±	4		81	±	2		48	±	1	#	52	±	2	#	84	±	4		88	±	5	
	C	90	±	6	*	87	±	5		47	±	4	#	56	±	3	#	89	±	5		95	±	6	
	N	83	±	5		78	±	5		42	±	2	#	51	±	2	#	84	±	4		81	±	4	
	I	85	±	3		83	±	1		44	±	2	#	52	±	3	#	84	±	4		89	±	5	
	C + N	88	±	2		81	±	2		47	±	2	#	48	±	2	#	89	±	3		89	±	3	
	C + I	88	±	6		86	±	6		50	±	2	#	56	±	4	#	91	±	6		95	±	6	
SV [ml]	control	24	±	1		24	±	1		15	±	1	#	16	±	1	#	27	±	1	#	27	±	1	#
	C	26	±	1		25	±	1		14	±	1	#	16	±	1	#	28	±	1		30	±	1	#*
	N	25	±	1		23	±	1		13	±	0	#	15	±	1	#	26	±	1		25	±	1	*
	I	25	±	2		24	±	1		13	±	1	#	15	±	1	#	27	±	1	#	29	±	2	#
	C + N	25	±	2		23	±	1		14	±	1	#	14	±	1	#	27	±	1		27	±	1	
	C + I	24	±	1		23	±	1		15	±	1	#	16	±	1	#	27	±	1	#	28	±	1	#
dPmax [mmHg^-1^·s]	control	432	±	24		400	±	22		332	±	22	#	324	±	12	#	392	±	11		428	±	24	
	C	486	±	17		452	±	19		334	±	21	#	362	±	25	#	418	±	30	#	454	±	34	
	N	474	±	21		416	±	39		322	±	25	#	330	±	27	#	404	±	41	#	434	±	49	
	I	486	±	29		464	±	29		326	±	28	#	340	±	25	#	418	±	37	#	428	±	35	
	C + N	476	±	14		428	±	20		300	±	10	#	342	±	17	#	414	±	20	#	436	±	25	
	C + I	482	±	23		450	±	21		358	±	19	#	376	±	22	#	476	±	64	*	514	±	59	*§
MAP [mmHg]	control	60	±	2		64	±	3		48	±	3	#	54	±	2	#	79	±	3	#	69	±	4	#
	C	62	±	2		64	±	2		50	±	2	#	58	±	2	#	82	±	3	#	74	±	3	#
	N	61	±	4		56	±	2		42	±	2	#	48	±	2	#	68	±	2	#	59	±	2	*
	I	59	±	2		60	±	2		47	±	2	#	54	±	1	#	79	±	3	#	67	±	5	#
	C + N	60	±	3		55	±	3		38	±	3	#	46	±	3	#	65	±	3		60	±	3	*
	C + I	60	±	1		62	±	3		46	±	1	#	55	±	2		80	±	1	#	67	±	3	#
HR [min^-1^]	control	114	±	2		115	±	2		109	±	3		112	±	2		103	±	3	#	108	±	3	#
	C	116	±	4		116	±	3		111	±	2		114	±	3		105	±	4	#	107	±	3	#
	N	112	±	4		114	±	3		110	±	2		114	±	2		107	±	3		109	±	4	
	I	116	±	4		116	±	3		111	±	2	#	117	±	4		104	±	4	#	102	±	4	#*
	C + N	116	±	4		118	±	3		110	±	2		114	±	2		110	±	3	#	111	±	3	#
	C + I	116	±	4	§	116	±	4		110	±	3	#	114	±	2	*	107	±	5	#	108	±	4	#§

Systemic oxygen delivery (DO2; [ml·kg-1·min-1]), systemic vascular resistance (SVR; [mmHg·l-1·min-1]), cardiac output (CO; [ml·kg-1·min-1]), stroke volume (SV; [ml]), index for cardiac inotropy (dPmax; [mmHg-1·sec]), mean arterial pressure (MAP; [mmHg]) and heart rate (HR; [min-1]) after a sole or combined local treatment with carbachol (C), nitroglycerine (N) or iloprost (I). Values are reported under baseline conditions (00:30), after drug application (01:00), during early (01:30) and late (02:00) hemorrhage and during early (02:30) and late blood retransfusion (03:00). The time is indicated as [hh:mm]. Data are presented as mean ± SEM for n = 5 female dogs. # p < 0.05 vs. baseline values; * p < 0.05 vs. control group; § p < 0.05 vs. a sole nitroglycerine or iloprost treatment, if carbachol was applicated simultaneously. 2-way ANOVA for repeated measurements followed by Bonferroni post-hoc test.

**Table 4:** Arterial blood gas analysis.

Variable	group	00:30				01:00				01:30				02:00				02:30				03:00			
S_a_O_2_ [%]	control	99	±	0		99	±	0		98	±	0	#	98	±	0	#	99	±	0		99	±	0	
	C	99	±	0		99	±	0		98	±	0	#	98	±	0	#	99	±	0		99	±	0	
	N	99	±	0		99	±	0		98	±	0	#*	98	±	0	#*	99	±	0		99	±	0	
	I	99	±	0		99	±	0		98	±	0	#	98	±	0	#	99	±	0		99	±	0	
	C + N	98	±	0		98	±	0		97	±	0	#*	98	±	0	#*	99	±	0		99	±	0	
	C + I	99	±	0		99	±	0		98	±	0	#	98	±	0	#	99	±	0		99	±	0	
p_a_O_2_ [mmHg]	control	151	±	3		150	±	3		146	±	3		146	±	4		156	±	4		154	±	4	
	C	147	±	2		149	±	4		144	±	4		145	±	3		158	±	2	#	158	±	3	#
	N	150	±	3		150	±	3		140	±	5	#	142	±	4	#	159	±	3	#	157	±	3	#
	I	152	±	2		156	±	2		145	±	4	#	147	±	3		160	±	2	#	160	±	2	#
	C + N	147	±	5		146	±	4		134	±	4	#*	144	±	5		162	±	4	#	161	±	5	#*
	C + N	148	±	3		152	±	4		143	±	3		144	±	2		157	±	4	#	157	±	3	#
p_a_CO_2_ [mmHg]	control	35	±	0		36	±	0		40	±	1	#	39	±	1	#	36	±	1		37	±	0	#
	C	35	±	1		35	±	1		41	±	1	#	39	±	1	#	36	±	1		36	±	1	
	N	36	±	1		36	±	1		42	±	1	#*	41	±	1	#*	37	±	0		37	±	1	
	I	35	±	1		36	±	1		41	±	1	#	40	±	1	#	36	±	1		36	±	1	
	C + N	36	±	0		37	±	0		43	±	1	#*	41	±	1	#*	37	±	0		37	±	1	
	C + I	35	±	1		36	±	1		41	±	2	#	40	±	2	#	36	±	1		35	±	1	*
pH	control	7.4	±	0.01		7.39	±	0.01		7.32	±	0.02	#	7.33	±	0.02	#	7.37	±	0.01	#	7.37	±	0.01	#
	C	7.4	±	0.01		7.39	±	0.01		7.31	±	0.02	#	7.31	±	0.02	#*	7.36	±	0.02	#	7.36	±	0.02	#
	N	7.39	±	0.01		7.38	±	0.01		7.3	±	0.01	#*	7.31	±	0.01	#*	7.35	±	0.01	#*	7.36	±	0.01	#
	I	7.4	±	0.01		7.39	±	0.01		7.32	±	0.02	#	7.33	±	0.01	#	7.38	±	0.01	#	7.38	±	0.01	#
	C + N	7.39	±	0.01		7.37	±	0.01	*§	7.29	±	0.01	#*	7.29	±	0.01	#*§	7.34	±	0.01	#*	7.35	±	0.01	#
	C + I	7.4	±	0.01		7.39	±	0.01		7.31	±	0.02	#	7.31	±	0.02	#§	7.37	±	0.01	#	7.39	±	0.01	*
HCO_3_^-^ [mmol·l^-1^]	control	21	±	0		21	±	1		20	±	1	#	20	±	1	#	20	±	0		20	±	0	
	C	21	±	0		21	±	0		20	±	0	#	19	±	1	#*	20	±	1	#*	20	±	0	#
	N	21	±	0		21	±	0		20	±	0	#	20	±	0	#	20	±	0	#	20	±	0	#
	I	21	±	0		21	±	0		20	±	0	#	20	±	0	#	20	±	0	#	21	±	0	
	C + N	21	±	0		21	±	0		20	±	0	#	19	±	0	#*§	19	±	0	#*	20	±	0	#
	C + I	21	±	0		21	±	0		20	±	0	#	20	±	1	#	20	±	0	#	20	±	0	#
Lac [mmol·l^-1^]	control	1.1	±	0.1		1.3	±	0.1		1.6	±	0.2		1.6	±	0.2		1.7	±	0.2		1.6	±	0.2	
	C	0.6	±	0.1		0.8	±	0.1		2.2	±	0.6	#	2.6	±	0.8	#*	2.4	±	0.7	#*	2.4	±	0.7	#*
	N	0.9	±	0.2		1.1	±	0.2		1.5	±	0.1	#	1.6	±	0.1	#	1.4	±	0.1		1.3	±	0.1	
	I	0.9	±	0.1		1.1	±	0.1		1.5	±	0.3	#	1.4	±	0.3		1.2	±	0.3		1.2	±	0.3	
	C + N	0.7	±	0.0		1.0	±	0.1		1.6	±	0.2	#	2.0	±	0.3	#	2.0	±	0.4	#	1.9	±	0.4	#
	C + I	0.9	±	0.0		1.2	±	0.1		2.2	±	0.6	#	2.4	±	0.6	#*	2.1	±	0.6	#	1.9	±	0.5	#
Hb [g·100ml^-1^]	control	12	±	0		12	±	0		12	±	0		12	±	0		12	±	0		12	±	0	#
	C	12	±	0		12	±	0		12	±	0	#	12	±	0		12	±	0	#	12	±	0	#
	N	12	±	0		12	±	0		13	±	0		12	±	0	*	12	±	0		12	±	0	#
	I	12	±	0		12	±	0		12	±	0		12	±	0		12	±	0		12	±	0	
	C + N	12	±	0		12	±	0	*§	12	±	0		12	±	0	§	12	±	0	§	12	±	0	#§
	C + I	12	±	0		12	±	0		12	±	0		12	±	0		12	±	0		12	±	0	#
Hct [%]	control	37	±	1		37	±	1		38	±	1		37	±	1		37	±	1		36	±	1	#
	C	37	±	1		37	±	1		38	±	1		37	±	1		36	±	1	#	36	±	1	#
	N	38	±	1		38	±	1		38	±	1		38	±	1	*	37	±	1		37	±	1	#
	I	37	±	1		37	±	1		38	±	1		37	±	1		37	±	1		37	±	1	
	C + N	37	±	1		36	±	1		38	±	1		37	±	1	§	36	±	1	§	36	±	1	#§
	C + I	37	±	1		37	±	1		38	±	1		37	±	1		37	±	1		36	±	1	#

Arterial hemoglobin oxygen saturation (SaO2; [%]), oxygen partial pressure (paO2; [mmHg]), carbon dioxide artial pressure (paCO2; [mmHg]), pH-values, bicarbonate concentration (HCO3-; [mmol · l-1]), lactate concentration (Lac; [mmol · l-1]), hemoglobin content (Hb; [g · 100ml-1]) and hematocrit (Hct; [%]) after a sole or combined local treatment with carbachol (C), nitroglycerine (N) or iloprost (I). Values are reported under baseline conditions (00:30), after drug application (01:00), during early (01:30) and late (02:00) hemorrhage and during early (02:30) and late blood retransfusion (03:00). The time is indicated as [hh:mm]. Data are presented as mean ± SEM for n = 5 female dogs. # p < 0.05 vs. baseline values; * p < 0.05 vs. control group; § p < 0.05 vs. a sole nitroglycerine or iloprost treatment, if carbachol was applicated simultaneously. 2-way ANOVA for repeated measurements followed by Bonferroni post-hoc test.

## 4 Discussion

This study investigated the effects of local carbachol alone and in combination with nitroglycerine or iloprost application on gastric and oral microcirculation and tissue oxygenation during hemorrhagic shock. According to literature, all substances might have a potential to induce microvascular recruitment. However, beneficial effects of local nitroglycerine and iloprost application might be partially counteracted by adrenergic vasoconstriction. Therefore, we investigated, if the simultaneous use of the parasympathomimetic carbachol leads to further microvascular recruitment and an improved tissue oxygenation in the gastrointestinal tract by reducing regional sympathetic effects. Since patients with hemorrhagic shock are highly vulnerable to additional adverse events due to the barely compensated hemodynamic situation, we chose local drug application to mitigate systemic side effects of the pharmacological treatment.

The main findings are:

(i) Local carbachol recruits the structural microcirculation of the oral mucosa, that leads to an increase of microvascular flow index in the late course of hemorrhage. Gastric but not oral tissue oxygenation improved during hemorrhagic shock.
(ii) Nitroglycerine application results in a potent increase of postcapillary oxygen saturation at the gastric mucosa, that cannot be enhanced further by an additional carbachol treatment. Local iloprost application slightly enhances gastric tissue oxygenation, that can be supplemented sufficiently by local carbachol treatment. Local drug application had no effect on oral microcirculation.
(iii) There are no systemic side effects on circulatory variables after local carbachol, nitroglycerine and iloprost or the combined application.

The main results of pharmacological treatment on gastric and oral mucosa are summarized in **Table 5** and are discussed in the following section. Local carbachol application improves gastric µHbO_2_ during hemorrhagic shock. Since µHbO_2_ mainly represents postcapillary oxygen saturation and the postcapillary region of the microcirculation had shown to be the area of risk under conditions of restricted oxygen delivery, an amelioration of gastric µHbO_2_ could indicate an increase of microvascular oxygen reserve after muscarinic stimulation (45). In general, there are three possible reasons for an increase of µHbO_2_ values. First, local drug application can induce a redistribution of circulating blood towards the investigated tissue with an increase of microvascular oxygen supply. µflow and rHb of the gastric mucosa remained unchanged over the time and pulmonary oxygen uptake was not restricted by an additional lung injury in this model of hemorrhagic shock. In consequence, microvascular oxygen supply did not increase after local carbachol substitution. Second, alterations of cell metabolism could lead to a reduced oxygen extraction fraction. Carbachol is reported to exert various effects on the physiological cell function of enterocytes. In detail, muscarinic stimulation leads to the phosphorylation of a Na^+^/K^+^-ATPase α-subunit (46) and liberates intracellular calcium (47). Both, α-subunit phosphorylation and an increased calcium concentration inhibit intestinal Na^+^/K^+^-ATPase-activity (47). Since maintenance of the electrical membrane potential is an extensively energy consuming process, inhibition of Na^+^/K^+^-ATPase-activity by carbachol could reduce cellular ATP demand and protect intestinal cells. Further, a slightly shifted electrical membrane potential and changes of the transmembrane ion gradients could influence basic cell metabolism as well. Since plasmatic lactate levels increased in all experimental groups that received carbachol treatment a reduced oxygen extraction fraction cannot only be explained by reduced cellular ATP demand, but also by cellular oxygen utilization disturbance. However, due to the minimal invasive access of the measurements and to avoid microcirculatory alterations after minor trauma related to tissue harvesting *in vivo*, we could not monitor mitochondrial function in this model of acute hemorrhage. Therefore, the reason and clinical relevance of the observed minimal increase of arterial lactate levels remains speculative. As the third possibility, changes of the structural microcirculation could increase gastric µHbO_2_ by two mechanisms, microvascular shunting and microvascular recruitment. Microvascular shunting separates tissue from a sufficient gas and nutrient exchange (48) and increases µHbO_2_ because of arterio-venous bypassing whereas inhomogeneous tissue perfusion and tissue hypoxia establish consequently over time. In critically ill patients and especially in patients with septic shock this phenomenon is observed frequently and seems to be one of the main microvascular derangements leading to organ failure and an increase of mortality (49). The significance of microvascular shunting and the formation of so called “weak units” (50, 51) in patients with hemorrhagic shock is still unclear. Contrary to microvascular shunting, pharmacological reopening of microvessels could reduce the oxygen diffusion distance from erythrocytes to stroma cells, reduce the total diffusion gradient and improve all over tissue oxygenation. Whereas microvascular shunting is associated with a decreased TVD and PVD, microvascular recruitment leads to an increase of both perfusion markers. In this study, oral TVD increased during hemorrhagic shock after local carbachol application indicating microvascular recruitment. This is additionally supported by our previous observation that improved oxygenation via nitroglycerine improved barrier function (25). In case of microvascular shunting the overall oxygen supply would be reduced and barrier function impaired. However, local carbachol treatment did not improve oral µHbO_2_ despite microvascular recruitment. Since oral µflow and PVD did not increase after topical carbachol treatment, we suggest that recruitment of oral vessels by carbachol application is accompanied by insufficient regional oxygen carrier supply during to hemorrhagic shock. Only MFI improved in the late course of hemorrhagic shock indicating favorable properties of carbachol on oral microcirculation. However, results from oral microcirculatory measurements cannot be transferred doubtless to other parts of the gastrointestinal tract, since our group could identify a different response of the oral and gastric microcirculation to topical melatonin treatment (36) and hypothermia (34) in a similar canine model of hemorrhagic shock. The observation that carbachol improves gastric but not oral oxygenation might indicate that during hemorrhage there is a more pronounced sympathetic activation in the oral region whereas the gastric region can still be modulated. As local modulation of the sympathetic and the parasympathetic nervous system is possible without systemic side effects, this seems a promising therapeutic option.

**Table 5:** Summary of microvascular effects of pharmacological treatment.

	Variable	µHbO_2_	µflow	µvelo	rHb	TVD	PVD	PPV	MFI
gastric mucosa	C	**↑** ^a^	**↔**	**↔**	**↔**				
	N	**↑↑**	**↔**	**↔**	**↔**				
	I	**↔**	**↔**	**↔**	**↔**				
	C + N	**↑** ^a^	**↔**	**↔**	**↔**				
	C + I	**↑** ^a^	**↔**	**↔**	**↔**				
oral mucosa	C	**↔**	**↔**	**↔**	↑ ^b^	**↑↑**	↑ ^b^	**↔**	↑ ^b^
	N	**↔**	**↔**	**↔**	↑ ^b^	**↔**	**↔**	**↔**	**↔**
	I	**↔**	**↔**	**↔**	**↔**	**↔**	**↔**	**↔**	**↔**
	C + N	**↔**	**↔**	**↔**	**↔**	**↔**	**↔**	**↔**	**↔**
	C + I	**↔**	**↔**	↑ ^b^	**↔**	**↔**	**↔**	**↔**	**↔**

Effects of local pharmacological treatment on gastric and oral microcirculatory variables during hemorrhagic shock are indicated using arrows. If only early (a) or late (b) shock was affected by sole or combined carbachol (C), nitroglycerin (N) or iloprost (I) treatment simple arrows (↑) were used. Beneficial effects during the whole course of shock were indicated by doubled arrows (↑↑). Incident dark-field imaging was performed only at the oral mucosa due to the minimal invasive instrumentation of the animals.

Summing up, muscarinic stimulation by local carbachol application leads to microvascular recruitment at the oral mucosa and improves perfusion quality. In this barely compensated vascular bed, microvascular recruitment by local carbachol application failed to improve regional tissue oxygenation possibly due to a remaining regional lack of microvascular tissue perfusion. At the gastric mucosa, local carbachol application enhances µHbO_2_ during hemorrhagic shock. From a mechanistic point of view, a change of the basic energy metabolism and microcirculatory recruitment could explain this effect of local carbachol application, but both mechanisms were not addressed in the experiments to avoid a more invasive instrumentation and microcirculatory alterations after minor trauma.

In a previous study, we could show that local nitroglycerine alone and iloprost alone improve gastric µHbO_2_ (25). Novel in this study is the effect of carbachol and the effect of the combination carbachol + nitroglycerine and carbachol + iloprost. The experiments with nitroglycerine alone and iloprost alone were conducted again as control group to generate an independent data set for this publication. Our recent findings are mainly in accordance with this previous data. Nitroglycerine enhanced gastric tissue oxygenation during hemorrhagic shock like described before, whereas local iloprost treatment led to slightly increased µHbO_2_ values compared to control animals in this study. However, the regime of local nitroglycerine and iloprost treatment was in accordance with our previous work. When local iloprost application was supplemented with carbachol, gastric µHbO_2_ further increased during hemorrhagic shock. A combined carbachol and nitroglycerine treatment did not reveal an additional effect on gastric tissue oxygenation. On the one hand, those findings could be related to direct effects of nitroglycerine, iloprost and carbachol on gastric microcirculation for example by a different potency to recruit gastric microcirculation. On the other hand, this could be the result of a subcellular crosstalk between two different signaling pathways. Muscarinic stimulation by carbachol application was reported to act via a nitric oxide dependent pathway (52). The combination of endogenous nitric oxide generation during hemorrhagic shock and an exogenous substitution of nitroglycerine could exceed the physiological range of available nitric oxide and lead to a maximal activation of nitric oxide related effects. If muscarinic effects exclusively depend on the nitric oxide pathway, this might explain why carbachol cannot further enhance gastric µHbO_2_ when applicated additionally to local nitroglycerine. Nevertheless, positive effects of nitroglycerine do not seem to be partially counteracted by autonomous neurogenic influence.

Prostacyclin effects within the microcirculatory compartment might be related at least in part to a different pathway that includes ATP release from erythrocytes (53, 54). The simultaneous activation of carbachol dependent nitric oxide generation and iloprost related ATP release, could lead to further improvement of gastric tissue oxygenation in incompletely recruited vessels. Additionally, iloprost activates an inosine triphosphate dependent pathway too, formatting a complex network of second messenger activation with various effects on regional homeostasis and cell metabolism. Furthermore, Herminghaus et al. already reported nitroglycerine and iloprost to improve the efficiency of oxidative phosphorylation in colon homogenates from healthy animals measured by respirometry (55). Therefore, downstream effects of local carbachol, nitric oxide and iloprost application could not only exert effects on microcirculation but influence mitochondrial respiration leading to an increase of gastric µHbO_2_. Since subcellular pathways of nitroglycerine, iloprost and carbachol have not been addressed in this study, further research should elucidate subcellular effects of the here used agents under conditions of restricted oxygen delivery.

Finally, drugs were applicated locally to avoid systemic side effects. In the past, our group used various local treatment regimens to improve gastric tissue oxygenation (34–36). None of those trials revealed systemic complications. In accordance, local carbachol application did not reveal systemic side effects on hemodynamic variables and macrohemodynamics were similar between all experimental groups, although we did not measure the intravenous concentration of carbachol in our experiments. During the experiments, we could not observe malignant arrhythmias like ventricular tachycardia or severe bradycardia. Even if local carbachol treatment was combined with either local nitroglycerine or local iloprost application there were no adverse effects on hemodynamic variables. Systemic lactate concentration increased in animals that received local carbachol treatment. However, as the measured values were only slightly higher than in the other experimental groups and plasmatic lactate concentration has many confounding factors, the physiological relevance of this finding remains questionable. Due to the reusable animal model the dogs were continuously under observation by the animal care staff that included veterinarians. Therefore, we state that local application of carbachol is safe and does not influence systemic homeostasis.

This gives the perspective to translate our approach into clinical studies. Local drug application to improve gastric microcirculation should be tested in patients with elective gastroscopy first. If local drug application emphasizes to be safe in healthy patients, the administration during interventional procedures and abdominal surgery should be tested in a second step. Patients with upper gastrointestinal bleeding appear to be the ideal patient population for transferring our results to humans, since these patients must be subjected to endoscopic hemostasis anyway. Local drug application could then follow acute bleeding control in the same session.

## 5 Conclusion

Local carbachol application induces favorable effects on oral microcirculation and gastric tissue oxygenation during hemorrhagic shock. Since microvascular perfusion remained unchanged after local carbachol supply, the down-stream effects of carbachol on cell metabolism and mitochondrial function could be interesting targets for tissue protection by local carbachol treatment during hemorrhagic shock and should be investigated in further studies. The effect of additional application of local carbachol to regional vasodilators depends on the combination used to improve microcirculation. Since different parts of the gastrointestinal tract seem to respond differently to local drug application, further studies should clarify, if the findings derived from this study can be translated to other parts of the gastrointestinal tract. However, local carbachol application and the combination with local vasodilators have no effect on the hemodynamic stability during hemorrhagic shock and therefore represent an interesting approach for tissue protection.

## Data availability statement

The original contributions presented in the study are included in the article/supplementary material. Further inquiries can be directed to the corresponding author.

## Ethics statement

The animal study was approved by Landesamt für Natur, Umwelt und Verbraucherschutz Nordrhein-Westfalen. The study was conducted in accordance with the local legislation and institutional requirements.

## Author contributions

SH: Data curation, Formal analysis, Visualization, Writing – original draft. LL: Data curation, Investigation, Writing – review & editing. MM: Data curation, Investigation, Writing – review & editing. CM: Writing – review & editing. AK: Writing – review & editing. AH: Conceptualization, Writing – review & editing. IB: Conceptualization, Project administration, Supervision, Writing – review & editing. OP: Conceptualization, Project administration, Supervision, Writing – review & editing. RT: Conceptualization, Project administration, Supervision, Writing – review & editing. CV: Conceptualization, Project administration, Supervision, Writing – review & editing.
